# Evaluation of internal target volume of abdominal tumors using cine‐MRI

**DOI:** 10.1002/acm2.70097

**Published:** 2025-05-13

**Authors:** Jessica Lye, Reza Alinaghi‐Zadeh, Sandie Fisher, Nikki Shelton, Glenn Cahoon, Leah McDermott, Richard Khor, Kym Rykers, Sweet Ping Ng

**Affiliations:** ^1^ Department of Radiation Oncology Olivia Newton John Cancer Wellness and Research Centre Austin Health Melbourne Australia; ^2^ School of Health and Biomedical Sciences RMIT University Melbourne Australia; ^3^ School of Molecular Sciences La Trobe University Melbourne Australia; ^4^ Department of Medical Imaging and Radiation Sciences Monash University Melbourne Australia; ^5^ School of Cancer Medicine La Trobe University Melbourne Australia; ^6^ University of Melbourne Melbourne Australia

**Keywords:** ITV, cine‐MRI, motion, MR‐Linac.

## Abstract

**Introduction:**

The detailed anatomy visualization with magnetic resonance (MR)‐guided radiotherapy is particularly attractive for abdominal treatments, but patient respiratory motion can compromise image quality. The “navigator technique” produces high‐quality 3D images, triggered by diaphragm displacement, in exhale phase only. The gold standard for planning is 4D imaging, which visualizes the lesion for all breathing phases. When 4D imaging is not available, an alternative is using 3D imaging combined with motion information from cine‐MR.

**Methods:**

This work investigates two alternative internal target volume (ITV) generation methods and compares them with the original treatment 4DCT imaging ITV. Datasets were analyzed from 10 upper abdominal patients that originally had been treated with a 4DCT‐based ITV. In addition to the 4DCT, these patients received an exhale MR and cine‐MR scans prior to treatment. An MR‐CT‐compatible motion phantom was also used to compare the two alternative ITV methods with the clinical 4DCT method. The first ITV method uses “margins expansion” (ME method) asymmetrically. The second method duplicates the exhale gross tumor volume (GTV) and shifts it to the positions of the average inhale GTV and mid‐position GTV. The ITV is the “Boolean combine” (BC method) of the three displaced GTVs. The ME and BC methods were compared with the clinical 4DCT method using the Dice similarity coefficient (DSC) to determine the impact of approximating the true GTV trajectory and neglecting deformation.

**Results:**

The ITV DSC ranges were 73%–96% for the ME method and 76%96% for the BC method. The BC approach created smaller treatment volumes than the ME method and more closely resembled the 4DCT margin for cases with larger motion and a significant component in the anterior–posterior direction.

**Conclusions:**

An exhale MR combined with cine‐MR can be used to simply create an ITV for adaptive MR‐guided radiotherapy. For small lesions with larger anterior motion, the Boolean Combine method is the more accurate method.

## 1 INTRODUCTION

Recent advances in linear accelerators with onboard magnetic resonance (MR)‐imaging and daily online adaptive workflows have greatly improved our ability to see and accurately target disease. MR datasets offer superb soft tissue contrast, but these advantages can be compromised by artifacts, including motion induced ghosting and blurring.^1^ The soft tissue contrast is especially advantageous over conventional CT for disease identification and normal tissue sparing of radiotherapy to the abdomen region, such as liver, kidney, and pancreas. However, these sites are particularly susceptible to artifacts during imaging due to respiratory motion. This presents challenges when accurately defining tumor displacement during treatment. Patient breath‐hold combined with treatment gating can minimize the impact of motion on treatment delivery, but not all patients can maintain a breath hold consistently. For these patients an alternative is to treat free breathing, with or without abdominal compression, using respiratory correlated CT (4DCT) or MR (4D‐MR) to create an internal target volume (ITV) that covers all breathing phases for treatment planning and simulation.

Currently, the Unity system from Elekta (Elekta, Crawley, UK) does not provide a 4D‐MR option for online adaptive radiotherapy. Paulson et al.^2^ used an in‐house high‐performance reconstruction server to generate the 4D‐MR datasets. These were then exported for parallel contouring sessions where the ITV was generated offline and subsequently transferred to online Monaco for adapted plan treatment. However, most MR‐Linac clinics do not have this option, and clinics can choose to use standard T1 or T2 for daily imaging (which may suffer blurring from motion artifacts), or to use one of the available motion management strategies. Three‐dimensional T2 navigated imaging captures the exhale phase by triggering acquisition according to the diaphragm position.^3^ T1 radial 3D gradient echo imaging, with “stack of stars” sampling (3D Vane), will capture the time‐weighted mean position with motion artifact suppression.^4^ Automated gating is a future option not yet available. When the navigated approach is used with free breathing patients, the gross tumor volume (GTV) is contoured only in the exhale phase and target motion must be accounted for with additional margins. Symmetric motion margins will over‐estimate dose required to the normal tissue superior to the target, or under‐estimate dose cover required inferior to the tumor, at the maximum inhalation phase.

Motion monitoring with high‐speed, single slice, cine‐MR images is another option available both prior to and during treatment. Stanescu et al.^5^ implemented a daily T2 navigator scan (exhale phase) for GTV contouring with ITV assessment from cine imaging for their online MR‐guided adaptation process. Akino et al.^6^ demonstrated the feasibility of creating an ITV from a GTV contoured on a mid‐phase 4DCT and generating a 3D trajectory (using Microsoft Visual C++ and OpenCV v2.2.0) from sagittal and coronal image sets. The method described in this work presents a simpler approach for ITV generation from cine‐MR, either through a simple margin expansion (ME) of the exhale GTV based on cine motion amplitude, or a Boolean sum or combination (BC) of the exhale, mid‐position, and inhale (translated) GTV. Cine sagittal and coronal image pairs provide motion assessment in all directions, but only in the selected 2D slice. Information on deformations and rotations, which are included with full 4DCT/MR imaging, will not be captured from the cine images, and so the impact of neglecting deformation is also assessed in this study. The position of the cine planes must be chosen carefully to ensure the target remains within the 2D slice for the full range of respiratory motion. The large slice thickness (5‐10 mm) of cine images is sufficient to capture most abdominal motion, but the corresponding volume averaging will reduce the accuracy of target edge visualization.

ITV generation from an exhale MR combined with cine mode motion assessment is useful for both pre‐treatment contouring and daily adaptation with recontoured GTV. Another emerging role for the use of cine imaging with MR‐Linacs is in the daily assessment of motion and the adaptation of margins if required. With routine 4DCT and ITV generation, there is an assumption that the respiratory motion captured at the initial planning phase is representative of the respiratory motion throughout the course of treatment. Interfraction changes in target motion can lead to lack of coverage or to excess radiation of normal tissue. Cusumano et al.^7^ compared a cohort of thoracic and abdominal patients with both planning 4DCT and continuous monitoring during the treatment with a sagittal cine‐MR. The thoracic lesions were all within the ITV for >95% of the time, while only 7 of 15 abdominal cases remained within the ITV for >95% of the treatment time. In a study by Fernandes et al.^8^ of liver and pancreatic lesions, it was observed that the breathing cycle from the 4DCT consistently underestimated the amplitude of motion compared to the cine‐MR that sampled over multiple breathing cycles. Other studies^9^, ^10^, ^11^, ^12^ came to a similar conclusion that a single cycle planning 4DCT does not adequately represent the daily intrafraction motion of abdominal tumors.

Fortunately, the increasing use of 4D‐MR will improve reference planning by accurately determining margins for the ITV. As well as improving tumor delineation in the individual respiratory phases, 4D‐MR also provides an improved representation of the range of motion amplitude across multiple breathing cycles. Chen et al.^13^ demonstrated that for 23 patients with primary liver cancer who had both 4D‐MR and 4DCT, the 4DCT underestimates the amplitude of motion. Even with reference 4D‐MR, the variation in motion between different treatment fractions remains as a source of uncertainty.

This study assesses the accuracy of creating daily adapted target volumes on the MR‐Linac with two ITV generation methods, using a daily exhale MR image. The first is based on asymmetric margins expanded from the exhale GTV, and the second combines the displaced GTV positions from cine‐MR imaging in the coronal and sagittal planes. Two methods of margin generation were tested with a phantom and then applied in a retrospective analysis to ten abdomen patients. The resulting target volumes were compared to the original clinical ITVs and planning target volumes, generated from target contours based on all breathing phases.

## 2 METHODS

### 2.1 Phantom planning target volume with cine mode

The Computerized Imaging Reference Systems (CIRS, Norfolk, USA) “Zeus” MR‐CT abdomen motion phantom was used to compare the ITV created from a 4DCT, to an ITV created using an exhale MR and cine‐mode imaging. Additionally, the PTVs generated with either a 3  or 5 mm margin expansion were compared. A large complex motion amplitude of 2 cm in the SI direction, 5 mm in the AP direction and 7 mm in the LR direction, with a cosine six waveform, was used to simulate large liver motion without abdominal compression.^14^ Two targets were investigated with this motion trajectory. The first used the target insert provided by the vendor to consider the extreme case of large motion amplitude on a complex shape with rotation (Case 1). The second target was derived from the 1 cm end of the cylindrical chamber insert, to consider the case of a small target with a large motion amplitude, but no rotational changes (Case 2). The amplitude for both cases was measured first with the cine image centered on the exhale phase, and subsequently centered on the inhale phase, to test motion assessment consistency.

**FIGURE 1:**
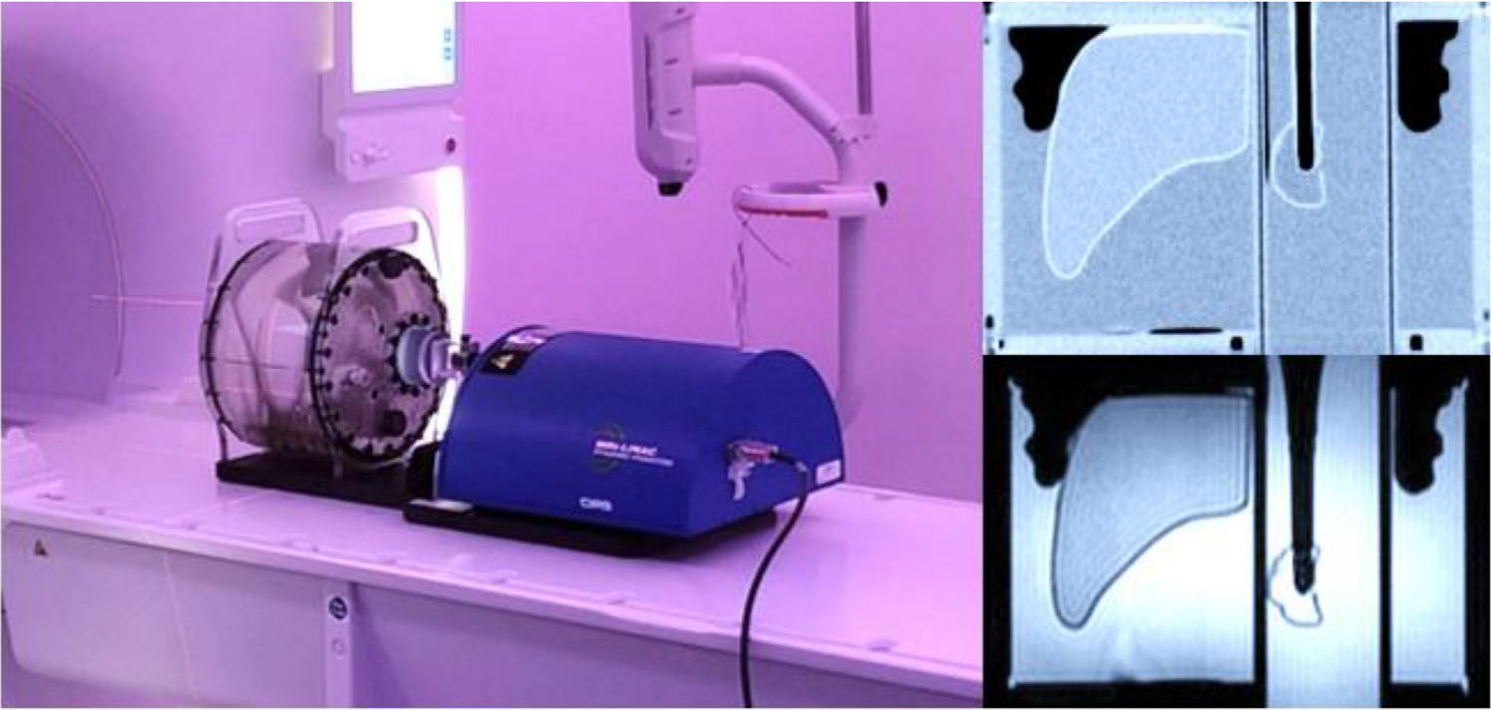
The motion abdomen phantom with CT (upper right) and MR 3D T2 (lower right) images of the target insert. MR, magnetic resonance.

The motion phantom (Figure 1) was imaged on a Siemens Somaton AS64 CT (Siemens, Erlangen, Germany). The VisionRT v3.0 (VisionRT, London, UK) system was used for phase binning of breathing phases to generate a 4DCT. The combined 4DCT image with all 10 phases, with a slice thickness of 2 mm, was exported to MIM Maestro (MIM software Inc, Cleveland, USA), where GTVs were contoured on each breathing phase and combined to create an ITV. The phantom was also imaged with an MRI using Phillips Ingenia Ambition 1.5T MRI (Philips, Best Netherlands). The sequences included a T2 3D with navigator, and balanced fast field echo (BFFE) cine mode, acquired in the sagittal and coronal planes. Imaging parameters of cine‐MR were TR/TE (repetition time/echo time) = 3.9/1.9 ms, 50° flip angle and 10 mm slice thickness. These are the sequences used for patient planning MR scans in our department.

**FIGURE 2:**
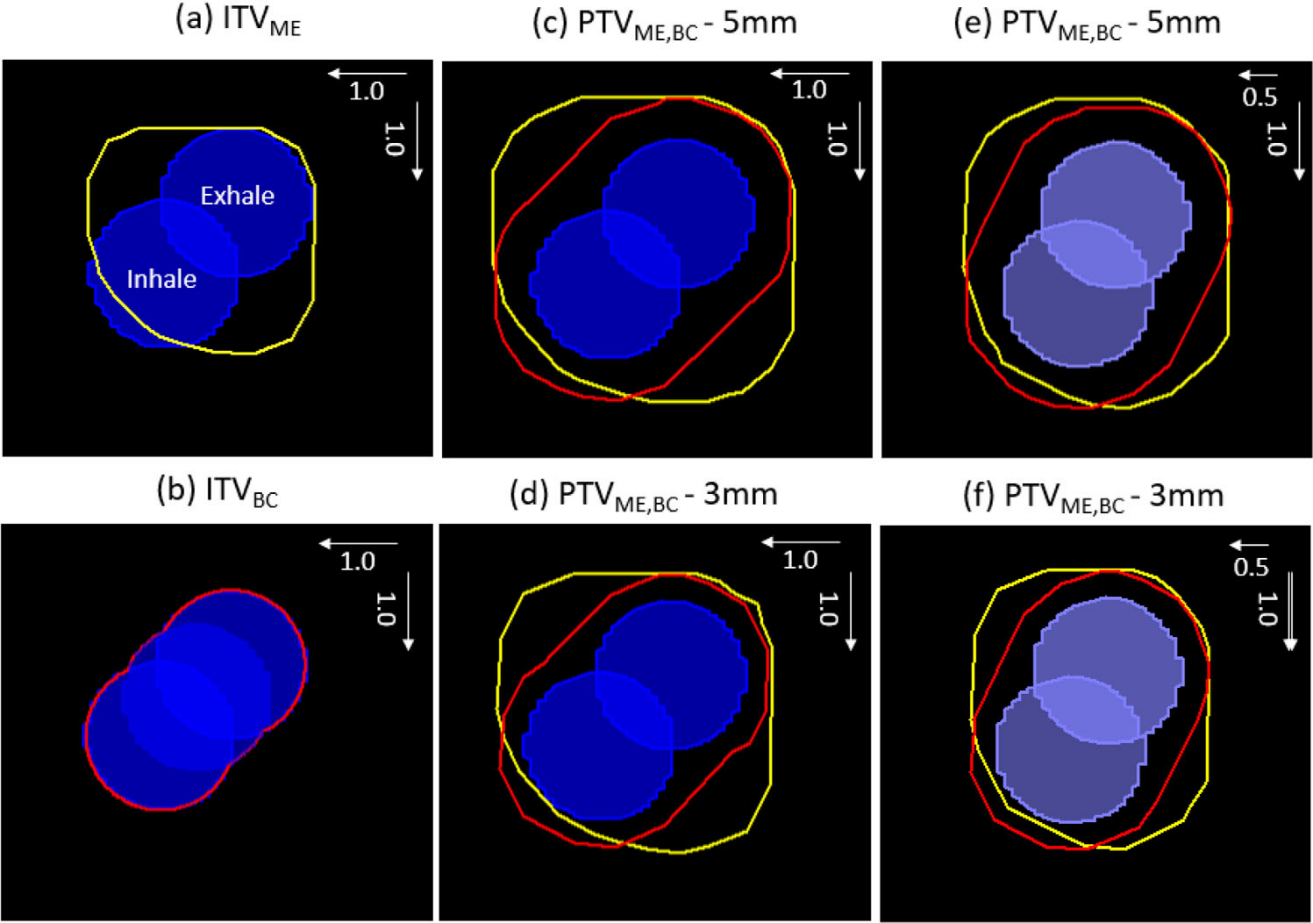
Illustration of the two methods for creating an ITV, and resulting PTVs, from an exhale image and set motion amplitudes from a sagittal view. (a) The GTV contoured at inhale and exhale, with a 1.0 cm motion displacement anterior and inferior. The ITV_ME_ (yellow) was created from the exhale phase with a margin expansion in the inferior and anterior directions. (b) The ITV_BC_ (red) created by duplicating and translating the exhale GTV to the mid‐ and inhale‐positions, then combining the three GTVs with a Boolean ‘OR’ function. (c–f) show the PTVs created from an isotropic expansion of the ITV_ME_ and ITV_BC_. (c) and (e) with a 5 mm PTV margin and 1.0 cm amplitudes, (d) and (f) with a 3 mm PTV margin, with a reduced anterior amplitude of 0.5 cm. GTV, gross tumor volume; ITV, internal target volume.

Two different methods were explored for creating an ITV from an MR, both using the exhale phase and measured motion amplitudes in orthogonal directions from cine images. The MIM Maestro software ruler tool was used to measure the maximum displacement. Figure 2 illustrates the two different ITV approaches, with both isotropic margin expansions, 3 and 5 mm, used to create a Planning Target Volume (PTV). The ITV_ME_ (yellow) is expanded from the exhale GTV in the inferior and anterior directions. The ITV_BC_ (red) is created by duplicating the exhale GTV and shifting it to the inhale position and the mid‐position, then combining the 3 GTV phases with a Boolean function tool. This method would be simple to implement as a MIM user workflow where the required motion shifts are entered inputs. Alternatively, the exhale GTV could be duplicated and moved to visually match the inhale and mid‐position shown on the cine, then combined with the standard Boolean function tool. Monaco has similar copy contour and Boolean contour combine tools. For online Unity Monaco, the inhale and mid‐GTV could be automatically generated from the online adapted GTV. In a second step, these generated GTVs could be manually shifted and combined to create the ITV_BC_.

Figure 2a–d show a 1.0 cm motion displacement in both the inferior and anterior directions. Figure 2e,f show the PTVs when the motion amplitude in the anterior direction is reduced to 0.5 cm. When using an orthogonal‐margin expansion to account for non‐orthogonal directions, a larger than required volume will be generated, which leads to both unwanted irradiation of normal tissue and a reduced dose to the target during the inhale phase. Even with the motion vector diagonal to the orthogonal directions, the GTV is just covered by the PTV margin. However, in (d) the exhale GTV expansion to PTV_ME_ barely includes the inhale GTV, indicating that coverage in this case may be compromised when the margin is determined by measuring the motion amplitude in the anterior and inferior directions. In theory, the PTV would be reviewed against the cine images, and coverage improved by increasing the margin in anterior and inferior directions. In practice, this would have a negative impact with excess normal tissue irradiated, as shown by the yellow PTV_ME_ in Figure 2c–f.

For the phantom study, the PTV_ME_ and PTV_BC_ were created from ITV_ME_ and ITV_BC_ respectively, using isotropic expansions of both 5 and 3 mm. These PTVs were compared to the 4DCT PTV using the contour compare tool in MIM Maestro. The dice similarity coefficient (DSC)^15^ and the mean distance to agreement (MDA) were evaluated along with the volume of each PTV.^16^

### 2.2 Patient planning target volume with cine mode

Datasets for 10 abdominal patients that had been planned free breathing on Elekta Versa linacs using an ITV approach were included in the study. These patients were unsuitable for spirometry‐assisted breath hold treatment gating. Images were acquired for 4DCT with the Siemens CT scanner and for MRI with the Phillips Ingenia MRI scanner. For the purposes of this comparative study, the 4DCT was used to verify the MR‐only method. In the proposed clinical solution, using the exhale 3D MR and MR‐cine, the 4DCT would not be required. All patient targets were visible on 4DCT and received a navigated 3D T2 MR and cine MR (in sagittal and coronal planes) prior to treatment. Patients were scanned for 30–60 s in cine mode, sufficient to capture at least five breathing cycles. The relatively short cine mode capture time was chosen to minimize the overall scanning time for the patient, with the trade‐off that only a small sample of breathing cycles are captured. Eight patients were imaged without abdominal compression and two with abdominal compression (ORFIT pressure belt, ORFIT Industries, NV, USA) as shown in Table 1. Patient 10 had a large motion amplitude and after an initial plan review was re‐scanned and re‐planned using 4DCT only (no MR), with abdominal compression. The motion amplitudes from Table 1 were obtained from the cine‐MR. This was compared to amplitudes from the 4DCT, which agreed to within 2 mm for most cases. For patient 1 and 6, the amplitude in the SI direction was 3 mm smaller when determined from the 4DCT. In all the cases in this study the amplitude in the LR direction was <2 mm.

**TABLE 1:** Lesion site, size, and motion amplitudes in each orthogonal direction, and whether the patient had abdominal compression.

Patient	Compression	Site	SI (cm)	AP (cm)	LR (cm)	GTV volume (cc)
1	No	Pancreas	0.8	0.5	<0.2	65.4
2	No	Adrenal	0.7	0.4	<0.2	4.1
3	No	Kidney	0.5	0.4	<0.2	229.0
4	No	Kidney	1.1	0.4	<0.2	62.0
5	No	Kidney	1.1	0.2	<0.2	123.4
6	Yes	Liver	1.1	0.7	<0.2	2.2
7	No	Kidney	1.1	0.3	<0.2	50.5
8	Yes	Pancreas	0.6	0.6	<0.2	25.6
9	No	Liver	0.3	0.3	<0.2	8.3
10^a^	No	Abdominal	2.2	1.0	<0.2	8.0

^a^ Patient subsequently replanned with abdominal compression

Abbreviation: GTV, gross total volume.

To create an ITV from the MR images, a rigid fusion was performed matching the clinical exhale GTV from the 4DCT to the corresponding target location in the MR exhale image. The motion amplitude was measured in MIM Maestro by using the ruler tool to measure the change in a fixed point on the target edge from inhale to exhale. The choice of target edge point was important as deformation of the apparent size and shape of the tumor can impact the measurement. The target edge was chosen where there was minimal change in apparent size of the tumor in the single slice cine between inhale and exhale. The motion amplitude was the mean of the amplitude over a minimum of five breathing cycles. The superior‐inferior amplitude was the mean amplitude over both the sagittal and coronal directions.

Irregular breathing refers to variations in the respiratory pattern of a patient undergoing imaging for radiation treatment planning. Unlike regular breathing, where inhalation and exhalation occur at consistent intervals and volumes, irregular breathing involves unpredictable changes in the depth or timing of breaths. The exhale baseline drift, as a measure of breathing regularity, was assessed across the five breathing phases. The midpoint between maximum and minimum exhale positions was used as the reference point. The patient exhale drift was within ±2 mm for all patients over the captured breathing cycles, and none of the patients were breathing irregularly. Abdominal compression may have helped achieve consistent exhale positions.

After the motion amplitude was determined, the two methods for creating the ITV shown in Figure 2 were then used to create ITV_ME_ and ITV_BC_. The PTV_ME_ and PTV_BC_ were subsequently created with a 5 and 3 mm isotropic expansion, for treatments on the Elekta Versa CBCT‐linac and Elekta Unity MR‐linac, respectively, according to our department clinical protocol.

These PTVs were compared to the 4DCT PTV using the contour compare tool in MIM Maestro. As for the phantom study, the DSC and the MDA were evaluated along with the volume of each PTV. It is expected that the largest impact of inaccuracies from margin expansions limited to orthogonal directions will occur when there is a significant component of motion in the anterior direction. The relative impact of the motion amplitude was estimated using a ratio of the motion to target size compared to the DSC and MDA results.

### 2.3 Respiratory phase identification

Additional analysis was performed to determine the respiratory phase capture of the T2 sequences on the Unity platform. Both phantom and patient 3D MR images were compared to the cine‐MR imaging, and the results are presented in the Supplementary Material.

## 3 RESULTS

### 3.1 Motion amplitude assessment

A potential limitation of assessing target amplitude with 2D cine image pair is the choice of imaging slice position, or plane, and the risk of the target moving outside of the chosen image plane. The accuracy of motion amplitude assessment was tested on the phantom using two cases as shown in Table 2, using a cine centered on either the inhale or exhale position. The first row shows the ground truth motion from the phantom programming. In all cases, the estimate of motion amplitude was within 2 mm of the expected value from the phantom programming.

**TABLE 2:** Measured motion amplitudes from the cine images.

	SI Sagittal (cm)	SI Coronal (cm)	AP Sagittal (cm)	LR Coronal (cm)
*Phantom motion*	*2.0*	*2.0*	*0.5*	*0.7*
Case 1 inhale cine	1.9	1.9	0.4	0.9
Case 1 exhale cine	1.9	2.0	0.7	0.7
Case 2 inhale cine	1.9	1.9	0.5	0.7
Case 2 exhale cine	1.9	2.0	0.5	0.7

*Note*: The first row shows the ground truth motion from the phantom programming.

### 3.2 4DCT target volume comparison with alternative ITV methods: ITV_ME_ and PTV_BC_

**FIGURE 3:**
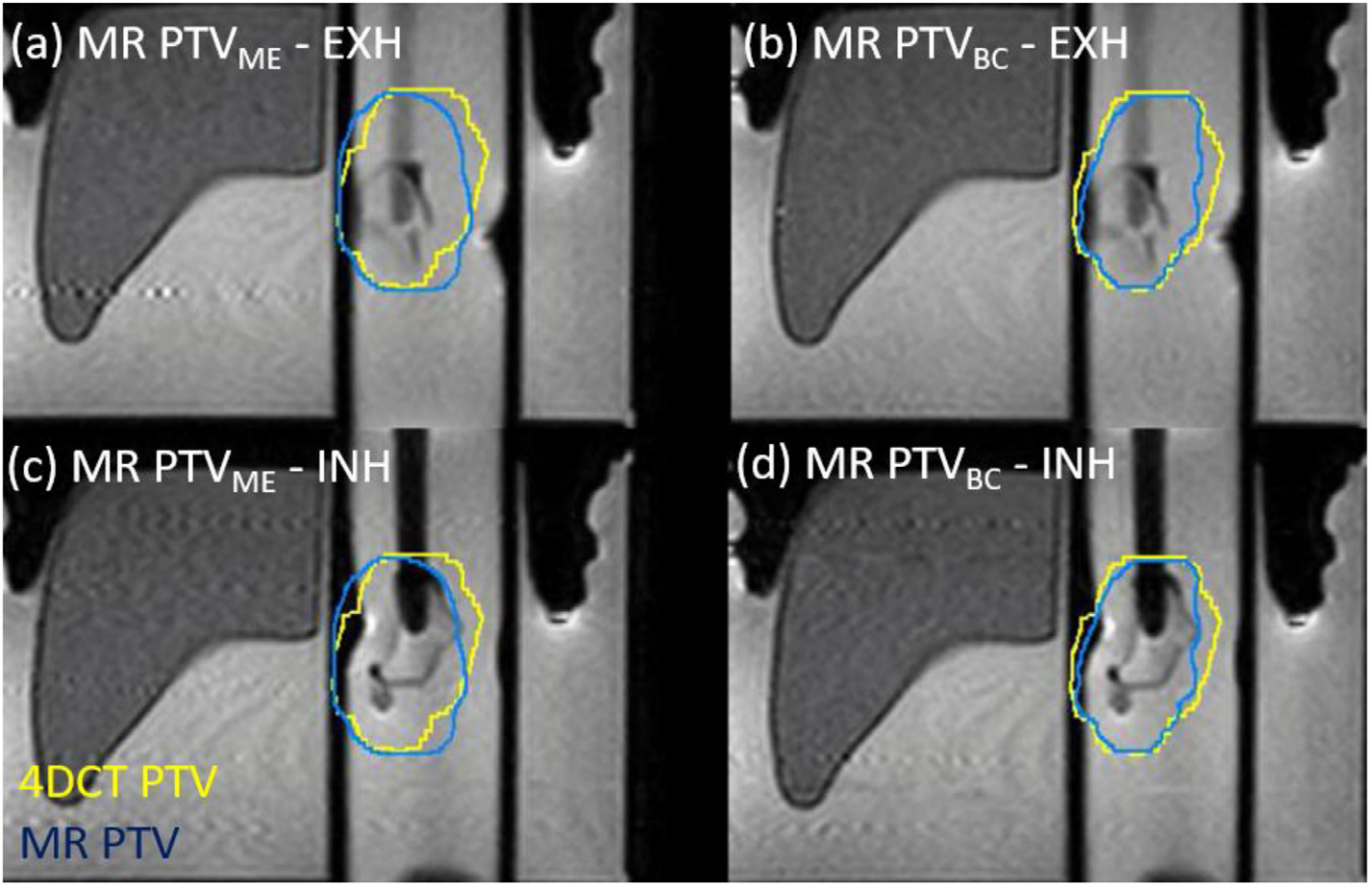
Comparison of phantom PTVs with a 5 mm margin created from the 4DCT (PTV_4DCT_, yellow), the MR exhale method (PTV_ME_, blue, left) and cine motion method (PTV_BC_, blue, right), on the phantom insert MR for case #PH1. (a) and (c) show the MR PTV_ME_ created with the margin expansion method compared to exhale and inhale cine image, respectively. (b) and (d) show the MR PTV_BC_ created with the Boolean combine method compared to exhale and inhale cine image, respectively. MR, magnetic resonance.

**FIGURE 4:**
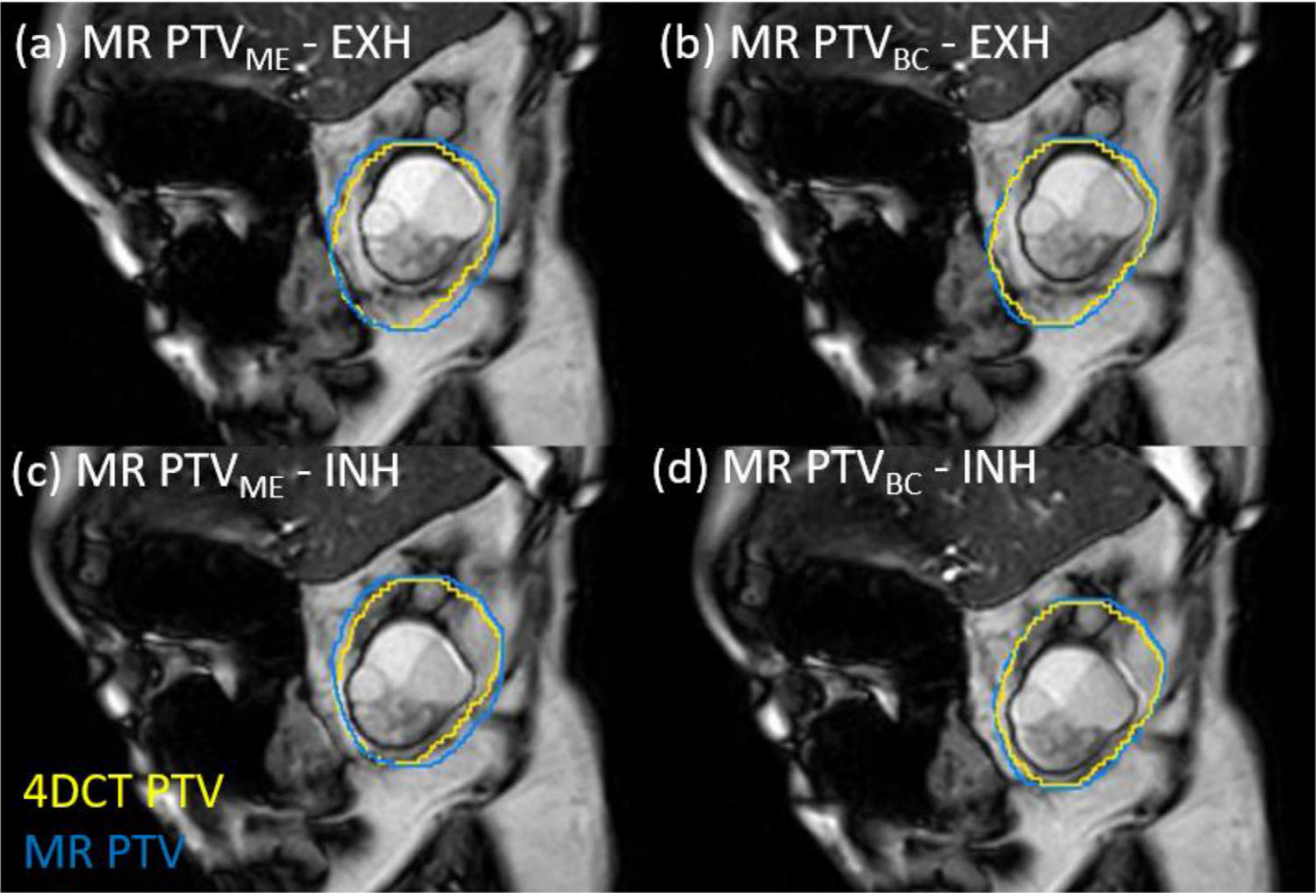
Comparison of patient PTVs with a 5 mm margin created with 4DCT (PTV_4DCT_, yellow), the MR exhale method (PTV_ME_, blue, left) and cine motion method (PTV_BC_, blue, right), on the patient MR for case #1, imaged on the Ingenia MRI. (a) and (c) show the MR PTV_ME_ created with the margin expansion method compared to exhale and inhale cine image, respectively. (b) and (d) show the MR PTV_BC_ created with the Boolean combine method compared to exhale and inhale cine image respectively. MR, magnetic resonance.

Figure 3 and Figure 4 show examples of the 5 mm PTV created for the phantom (Case #PH1) and patient (Case #1), respectively. Both figures show the target in the exhale phases (upper row) and inhale phases (lower row). The yellow contour shows the PTV from the full 4DCT information dataset. The blue contours show the PTV created from the margin expansion method, using the cine‐MR images (PTV_ME_, left column) and from the Boolean combine method, based on an exhale MR (PTV_BC_, right column).

In the phantom case (Figure 3), the exhale images are inverted with the exhale target in an inferior position as shown in the top row. The simulated motion curve was reversed in the SI direction so that more time is spent in the inferior position (inhale) than in the superior position (exhale). This was chosen to improve the navigated trigger, which was simulated using the end of the motion cylinder as a surrogate for a liver‐lung interface. It can be noted in both examples that the margin expansion method (PTV_ME_) results in a slightly wider planning volume, due to the diagonal component of the motion, with respect to the orthogonal axes in which the amplitudes are measured.

In the example phantom case shown in Figure 3, the DSC and MDA are 76% and 3.5 mm, respectively, for the margin expansion method (a) and (c). This improves slightly to 79% and 2.8 mm with the Boolean Combine method (b) and (d), but the target is almost touching the PTV in the inverted inhale phase, suggesting insufficient coverage. With a 3 mm margin expansion from ITV to PTV the DCE agreement reduces further to 73% and 76% for the ME method and BC method, respectively, and the target moves outside the PTV. This is due to the extreme condition simulated in the phantom case with a large rotation from a complex shape that is not likely to be observed clinically. In the patient example shown in Figure 4, the DSC and MDA are 89% and 2.0 mm, respectively, for the margin expansion method (b) and (d). This improves slightly to 93% and 1.2 mm with the Boolean Combine method (a) and (c). With a 3 mm margin expansion from ITV to PTV, the DCE reduces to 87% and 92% for the ME method and BC method, respectively.

**FIGURE 5:**
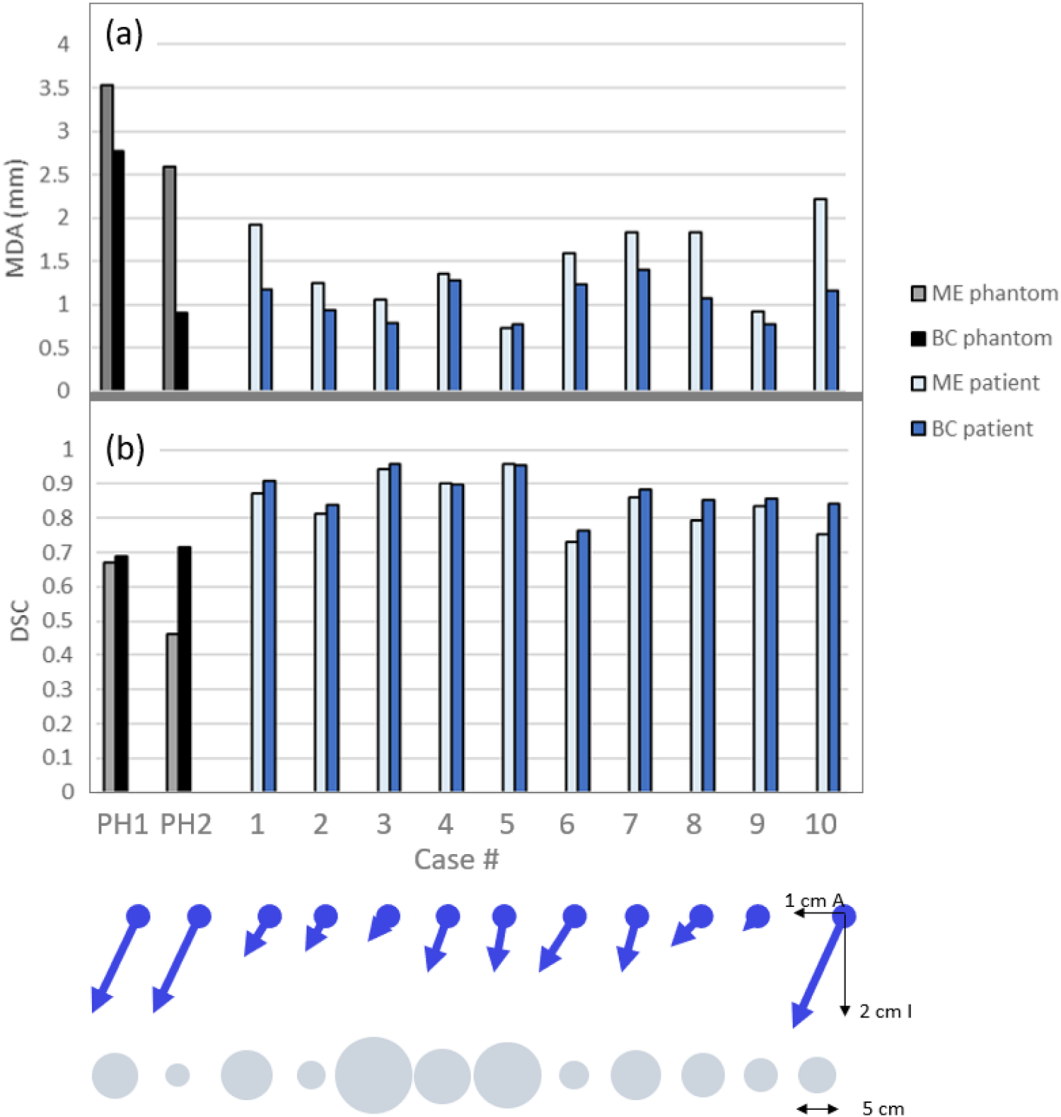
(a) The MDA and (b) the DSC, comparing the ITV_4DCT_ to the ITV_BC_ and ITV_ME_. Shown below the chart is the motion amplitude in the interior and anterior directions, and the relative (scaled) diameter size of each lesion. DSC, Dice similarity coefficient; ITV, internal target volume; MDA, mean distance to agreement.

For both the phantom and the patient cases, the DSC and the MDA were evaluated by comparing the ITV_4DCT_ to either the ITV_ME_ or ITV_BC_, as shown in Figure 5a,b. For the ITV_BC_, the patient case 6 DCE was 76.1%, while the DCE for all other patient cases was greater than 80%. For the ITV_ME_ method, patient case 6 and 10 were below 80% with DSC values of 72.9% and 75.2%, respectively. Phantom case 2, with a small volume and large diagonal motion amplitude, was particularly problematic for the margin expansion method, with a DSC of only 46.2%.

**FIGURE 6:**
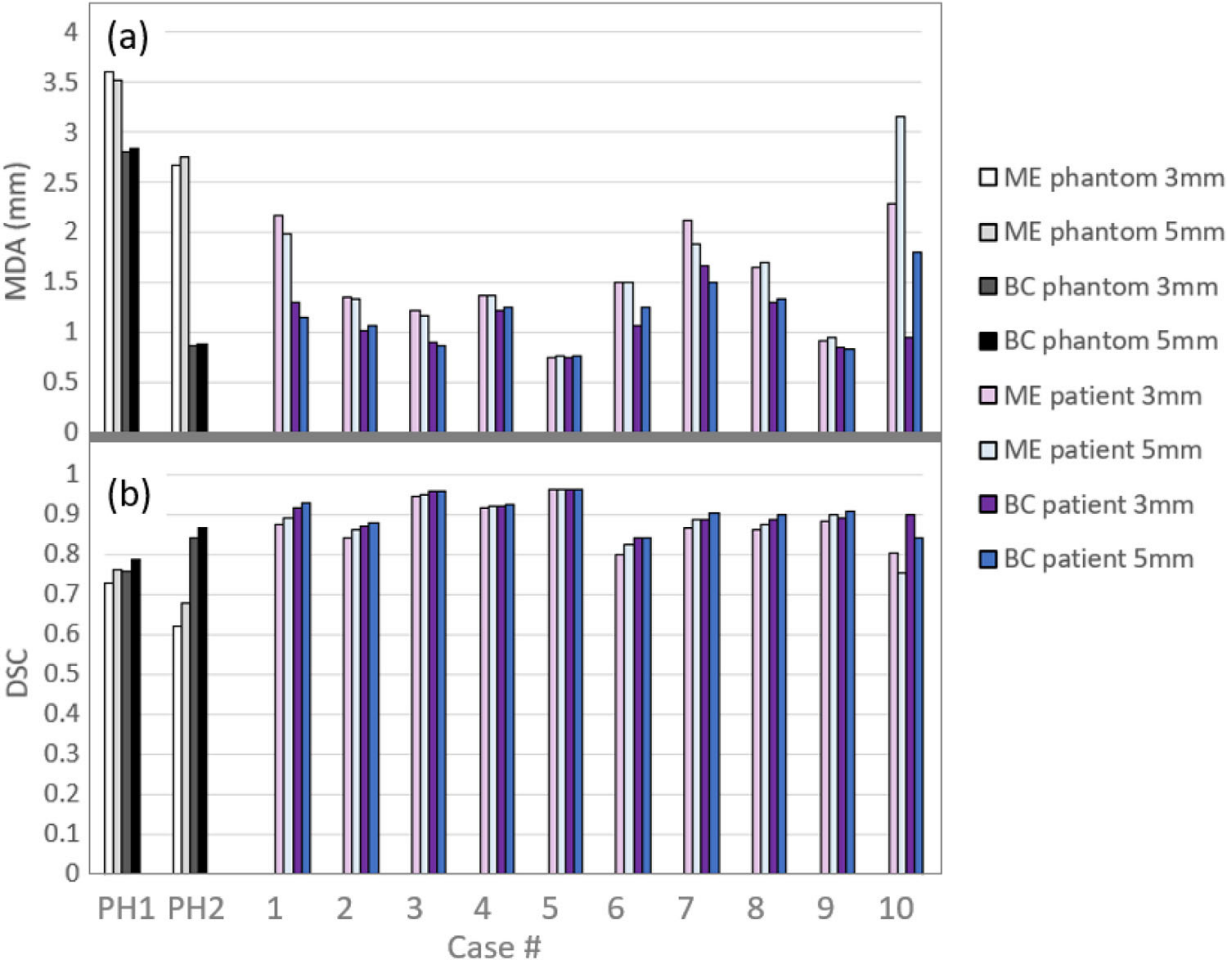
(a) The MDA and (b) the DSC, comparing the PTV_4DCT_ to the PTV_BC_ and PTV_ME_, created with 3 and 5 mm margins. DSC, Dice similarity coefficient; MDA, mean distance to agreement.

The DSC and the MDA were also evaluated for the PTVs with a 3 and 5 mm margin expansion as shown in Figure 6. Figure 6a,b show the DSC and MDA, respectively, comparing the PTV_4DCT_ to PTV_BC_ and PTV_ME_. Both alternative MR methods show high levels of agreement with the 4DCT PTV method, where the DSC is >80% for most patient cases, except for the PTV_ME_ of patient 10 (75.4% and 80.3% for 5 and 3 mm margins, respectively) and patient 6 (82.5% and 79.7% for 5 and 3 mm margins, respectively). The PTV_BC_ for phantom case 1 had a DCE < 80% (78.5% and 75.7% for 5 and 3 mm margins, respectively). In this case the source of the difference could be attributed to the large rotation of a complex target shape, with a degree of rotation that is not seen in clinical scenarios. For the margin expansion method, larger volume differences were observed for the PTV_ME_ when the lesion was small, and the motion had a large anterior component (phantom case 2 and patient cases 6 and 10). However, even for these scenarios, the patient case DSC remained above 75%. For all the patients in this study, the left‐right GTV motion component was very small. It is expected that similar behavior would be seen for patients with significant left‐right motion. When the motion causes the target trajectory to move in non‐orthogonal directions, the DSC reduces.

**FIGURE 7:**
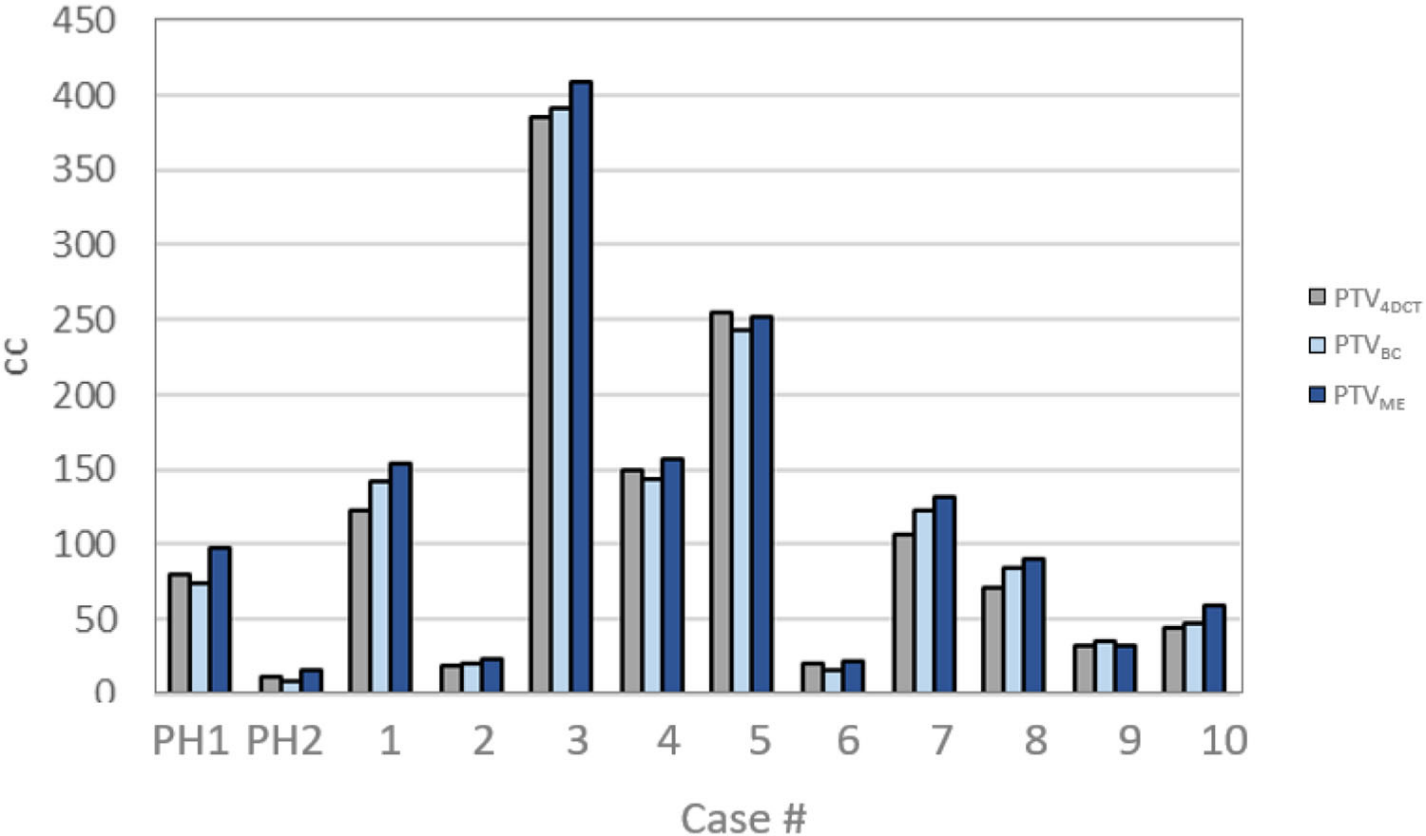
Comparing the volume of the PTV_4DCT_, PTV_BC,_ and PTV_ME_ for the phantom and patient cases using a 5 mm margin expansion.

The change in planning volume when using the two methods for creating the MR based PTV with a 5 mm expansion is shown in Figure 7. In all cases the PTV_ME_ had the largest volume with the largest difference to PTV_4DCT_ of 35% for case 5. For the PTV_BC_ method, the largest difference was 15%, seen for case 3 due to slightly larger amplitude measured on the cine imaging compared to the 4DCT.

**FIGURE 8:**
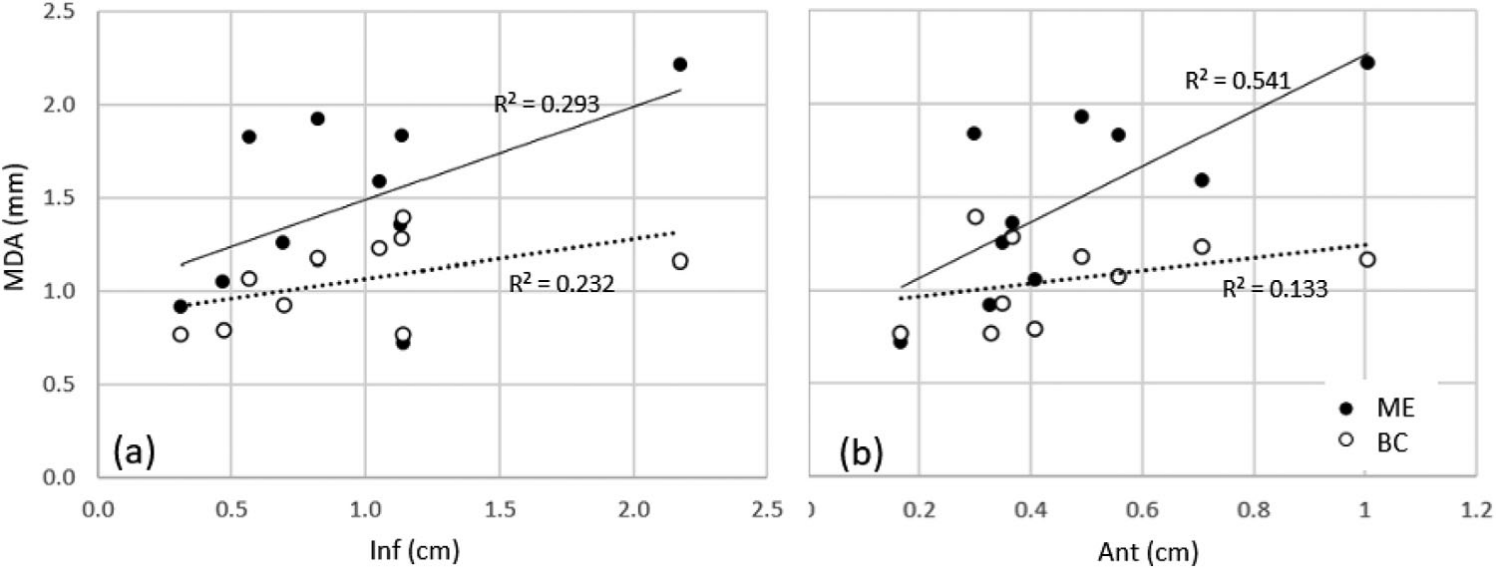
The correlation between MDA of the ITV comparison using the Margin expand or Boolean Combine method and motion in (a) the inferior and (b) anterior direction. ITV, internal target volume; MDA, mean distance to agreement.

**FIGURE 9:**
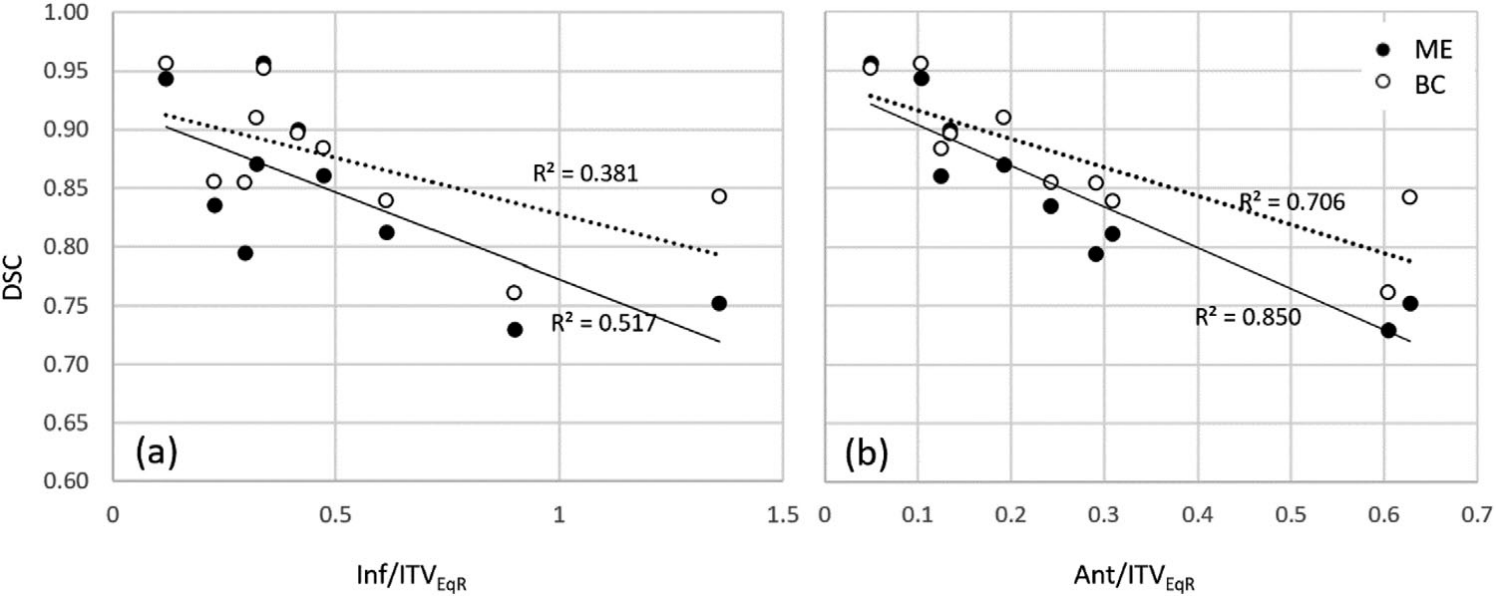
The correlation between DSC of the ITV comparison using the Margin expand or Boolean Combine method and the ratio of (a) the inferior motion to the ITV equivalent radius and (b) the anterior motion to the ITV equivalent radius. DSC, dice similarity coefficient; ITV, internal target volume.

The correlations between the inferior and anterior motion and the MDA and DSC metrics were investigated. Figure 8 shows the correlation between the MDA of the ITV comparison using the Margin Expand or Boolean Combine method and motion amplitude. The highest correlation was seen using the Margin expand method, with the MDA increasing with the anterior motion Figure 9 shows the correlation of the DSC to a ratio of motion to ITV equivalent spherical radius (ITV_EqR_). The strongest correlation was seen with anterior motion, where the DSC dropped below 75% when the anterior motion was approximately half the equivalent radius.

## 4 DISCUSSION

This study showed the potential of using orthogonal cine‐MR motion amplitude information, together with a navigated exhale cine‐MR image, to create an ITV for free breathing, non‐gated, upper abdominal radiotherapy. The methods tested in this work provide a pathway to create accurate treatment volumes when using navigated daily MR images, to definitively capture a known respiration phase, and avoid unnecessary irradiation of normal tissue or incomplete coverage of the target.

The approach of using cine‐MR with an exhale MR to create an ITV was explored by Akino et al. where in‐house code was developed to calculate the motion vectors between adjacent frames of the cine, which were then used to propagate the target contour.^6^ For the six patients with hepatocellular carcinomas investigated by Akino et al., the DSC ranged from 70%–90%, comparing the cine‐MR ITV to the ITV 4DCT, depending on the patient and the potential cut‐off value. Two simpler methods were investigated in our study, using either an asymmetric margin expansion from the exhale phase, or translating the exhale GTV to the mid‐ and inhale‐positions and then combining the three GTVs. The DSC for the eight patients in this study ranged from 73%–95% for the margin expansion method, and 76%–96% for the Boolean combined method. Both methods were simple to implement offline and achieved similar DSC results to the motion vector translation approach in ref. [6] The Boolean combine approach created smaller treatment volumes than the asymmetric margin expansion method and was more accurate when there was a large motion amplitude component other than in the superior‐inferior direction. For these types of lesions, the additional step of duplicating and shifting the GTV may be the preferred approach, however this would require additional manual steps. Software could be developed to replicate the manual steps required for the purpose, or the process could be created as an automated workflow in third‐party software such as MIM.

Previous work^17^ investigated various planning strategies for lung cancer treatment and concluded that the ITV concept over‐estimates the margin required for respiration motion, compared to a mid‐ventilation (midV) approach. The midV approach has also been used for SBRT in upper abdomen treatments,^18^ to reduce normal tissue irradiated. Van de Lindt et al.^19^ proposed using a midV approach with two interleaved navigator triggered MR images, one in exhale and one in the midV position, and using in‐house code to deform the high‐resolution exhale to the mid‐position. The exhale MR and cine motion assessment used in this work could be extended to a midV‐like approach either by shifting the GTV to the midV position or use of an asymmetric margin recipe.

A limitation of using only an exhale image with the cine MR motion assessment is that rotation, deformation and non‐linear motion trajectory are not accounted for.^20^, ^21^, ^22^, ^23^ Furthermore, careful placement of the cine slice is important to ensure small targets do not move outside of the imaging plane. Phantom assessment of motion when the cine slice was centered on the exhale position versus the inhale position yielded results within 2 mm of the actual motion. Uncertainty in the motion assessment will also occur if the target or surrogate edge is deforming during the respiration cycle. Despite these limitations, with the small sample of clinical cases investigated in this study, the ITVs created showed good agreement with the full 4DCT assessment, even with large motion amplitudes of approximately 2 cm in the cranial–caudal direction.

The clinical cases investigated in this work were chosen with visible lesions in both the 4DCT and the cine‐MR images. Coverage can be difficult to determine in cases where the lesion is not clearly visible in the cine‐MR images but are visible in the 3D exhale MR. In these cases, surrogates such as the liver edge can be used to assess the motion, however the coverage from the margin expansion method may not be sufficient. Breathing motion can become irregular during treatment, and motion monitoring during MR‐guided treatment is used to check for any such changes in breathing. Treatment can be interrupted by the therapist if irregular breathing causes the target to move outside the PTV. If the initial treatment margins determined during simulation are insufficient for target coverage during treatment, the ITV margins can be increased using the motion monitoring MR‐cine information.

The aim in this work was to assess two methods for generating the best PTV in an online workflow, as shown by the DSC and MDA values. This work provides indications for which patients the BC method is an improvement over the simpler ME method based on motion vectors and target size. The results of this study could inform a full optimization and replanning comparison of PTV dose over multiple fractions across an adaptive workflow in a future study.

An advantage of using the cine‐MR is the high‐temporal resolution and ability to assess the breathing motion over many respiratory cycles and across multiple days or fractions. Cine‐MR also does not contribute additional dose to the patient and can be used for repeated image acquisition. The single respiratory cycle captured in 4DCT may underestimate the amplitude of the motion over many breathing cycles.^8^ The respiratory motion can change over the course of treatment and can also be impacted by the abdominal compression of the day.^9^, ^10^, ^11^, ^12^ The method in this work could be used for daily adaptation of the motion amplitude margins to further optimise MR‐guided, free breathing radiotherapy.

## 5 CONCLUSIONS

This work investigated two alternative ITV generation methods based on an MR exhale and MR‐cine images and compared them with the original treatment ITV from 4DCT imaging. These ITV methods are applicable to daily ITV creation on an MR‐Linac or in simulation when 4D‐MR is not available. The study demonstrates that an exhale MR, combined with cine motion information, can be used to simply create an ITV, using either a non‐isotropic margin expansion or boolean combine of the maximum GTV displacement. For lesions with a significant anterior–posterior component, the Boolean combine method may be the preferred approach, although this would require additional software development for efficient implementation for online adaptation. The techniques also offer the possibility of daily adaptation of treatment margins to further optimise MR‐guided free breathing radiotherapy.

## AUTHOR CONTRIBUTIONS

All authors made significant contributions to one or more of the design, preparation, and execution of the study, including experimental design, data acquisition, analysis, interpretation, manuscript drafting, and review.

## CONFLICT OF INTEREST STATEMENT

The authors declare no conflicts of interest.

## Supporting information



Supporting Information
