# Supplementary material for: Evaluation of internal target volume of abdominal tumors using cine‐MRI
Source: J Appl Clin Med Phys. 2025 May 13;26(6):e70097. doi: 10.1002/acm2.70097 (PMC12148771; doi:10.1002/acm2.70097)
Supplement: Supplementary file 1 — Supporting Information [file ACM2-26-e70097-s001.docx]

Supplementary materials

## Respiratory phase identification

The identification of the respiratory phase captured in each 3D MR sequence used for daily image guidance is critical for accurate targeting. The Computerized Imaging Reference Systems (CIRS, Norfolk, USA), “Zeus“ MR-CT abdomen phantom was used with different breathing waveforms (patient recorded and synthetic) to test the respiratory phase capture on the Unity system. The phantom was imaged with the Elekta recommended MRI T2 3D sequences for abdomen, both with and without the navigator on the Unity Marlin 1.5T MRI. Balanced fast field echo (BFFE) sequences acquired in cine mode from the sagittal and coronal planes was used to evaluate the imaging phase and motion amplitude of the phantom. All image evaluation was performed in MIM v7.1.6 Maestro software (MIM Software, Cleveland, OH). MRI 3D T2 images taken of patients on the Unity system with and without the navigator were also compared to cine mode images, to determine the captured respiratory phase. Ten structures in the upper abdomen region from five patients were analysed, their details are listed in Table 1.

Table 1 List of the structure motion in superior-inferior direction for five patients imaged with the Unity MRI T2 3D sequence, both with and without the navigator.

| Patient | Compression | Site | Structure1  (cm) | Structure2  (cm) |
| --- | --- | --- | --- | --- |
| A | Yes | liver | 0.5 | 0.7 |
| B | Yes | liver | 0.6 | 0.4 |
| C | Yes | pancreas | 0.8 | 0.4 |
| D | Yes | pancreas | 0.5 | 0.4 |
| E | Yes | abdominal | 0.8 | 0.9 |
|  |  |  |  |  |

Figure 1 shows phantom images from using two different types of patient breathing waveforms scaled to create a 10 mm peak-to-peak motion amplitude in the superior-inferior direction only. The phantom insert was imaged on the Unity Marlin MRI using the T2 3D in the exhale position with no motion (a) and (d), with the T2-navigated scan including motion (b) and (e), and non-navigated scan with motion (c) and (f). An ITV was created from the exhale T2 without motion and the sagittal cine image, using the ‘duplicate, shift to inhale and combine’ method. This is shown by the green contour. In this phantom test, there is no deformation or rotation, and motion is solely in the superior-inferior direction, so the ITV is an accurate representation of the GTV location over the average breathing cycle. The apparent inferior edge of the target in the cine image is compared to the T2 3D image in the exhale images to ensure it is not shifted. In the lower series of images in Figure 1, the phantom respiratory motion produces a saw-tooth pattern, while in the upper series of images the phantom respiratory cycle spends more time in the exhale phase. A review of the phantom images shows that the T2 3D-navigated images provide a clear image in the exhale phase for both types of breathing waveforms. There is no shift in the navigated image within the uncertainty of the 1.2 mm slice thickness. This was evaluated by the centroid position of a rigid shift of the exhale contour from the still image to the navigated image. Without the navigation, the T2 3D imaging with the upper breathing patient waveform result in slightly noisier images but still shows the target in the exhale phase. When the patient breathing follows a saw-tooth pattern, the T2 3D images become blurrier and appear in the time-weighted average position. The standard T2 sequence uses the serial motion artefact reduction technique (SMART) where the initial measurement is made for all values of the phase encoding before repeat measurements are commenced. The SMART option is used in both the navigated and non-navigated sequence. The separation in time of data acquisition reduces the appearance of the motion artefact with non-symmetrical breathing patterns and the image appears to be in exhale rather than the time weighted average without navigation as shown in Figure 1 (c). This behaviour was also investigated with synthetic Sin and Cos6 breathing traces and similar behaviour was observed.


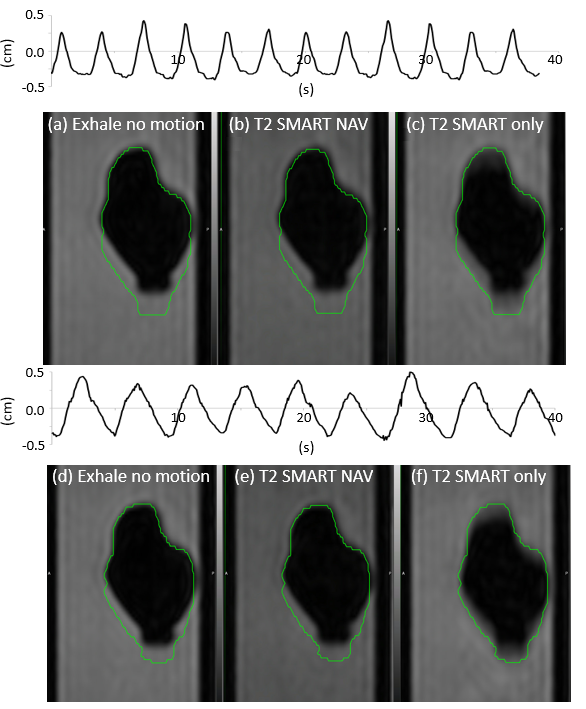


Figure 1 The phantom insert was imaged with 3D T2 without motion in exhale position (a) and (d), and with motion T2 using navigation and SMART (b) and (e), and with motion with SMART only (c,f). (a-c) shows the series of images with the top respiratory trace and (d-f) with the lower respiratory trace. The ITV contour is shown in green.

Patient 3D T2 images (with SMART averaging) from the Unity Marlin MRI were compared to the sagittal cine-MRI images to determine the respiratory phase capture. The 3D T2 images were acquired with and without the navigator. Ten structures (either GTV or clearly visible regions of interest) in the upper abdomen region from five patients were analysed. The average distance from the inferior and superior structure edge contoured in the 3D T2 image to the structure edge in 10 inhale and exhale phases of cine-MRI was measured in the MIM software. For the navigated images all structures were in the exhale phase. The results are shown in Figure 2 for the non-navigated images. The majority of the structures in the 3D T2 images align with the exhale respiratory phase of the cine-MRI image as shown by a zero shift. There was one clear exception (Patient E), where the structure was midway between the exhale and inhale phase. This example is shown in more detail in Figure 3, where the exhale and inhale from the BFFE cine mode is compared to the T2 with and without navigation and the T1 image. The ITV contoured from the cine mode is also shown as a visual guide. This patient had rapid breathing motion with a saw-tooth pattern whereby similar time was spent in inhale and exhale, and SMART was unable to correct to the exhale phase. For this patient the navigated scan was required to capture an exhale phase. It is not safe to assume that a non-navigated T2, even with SMART averaging, will show the exhale phase for all patients.


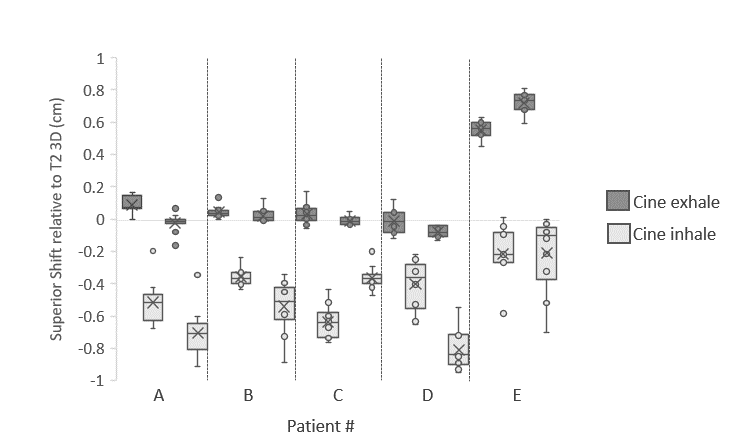


Figure 2 The average distance from the inferior and superior structure edge contoured in the 3D T2 image to the corresponding structure edge in ten inhale and exhale phases of cine-MRI for the non-navigated MRI images.


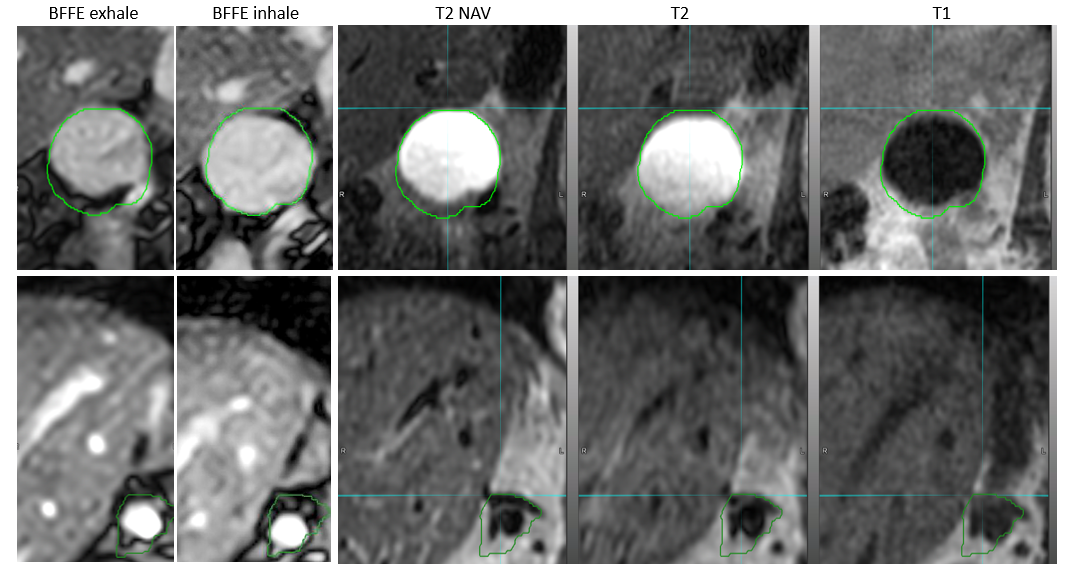


Figure 3 The visible ROI is shown in the exhale and inhale phase of the cine-MRI movie, with the ITV contoured in green. The ITV contour from the cine-MRI is shown on a navigated T2, and a non-navigated T2 image for patient E
